# Thrombophilia Testing: from Genetic Predisposition to Discrimination

**DOI:** 10.1055/a-2284-4285

**Published:** 2024-04-08

**Authors:** Andreas Verstraete, Kathleen Freson, Peter Verhamme, Thomas Vanassche

**Affiliations:** 1 Department of Cardiovascular Diseases, University Hospitals Leuven, Leuven, Belgium; 2 Department of Cardiovascular Sciences, Center for Molecular and Vascular Biology, KU Leuven, Leuven, Belgium

Venous thromboembolism (VTE) is a multifactorial disease arising from a complex interplay between acquired clinical risk factors and inherited genetic predispositions. VTE is a common condition and occurs in 1 to 2 per 1,000 people every year in Western countries. 1 2 Laboratory tests for inherited thrombophilia are frequently requested with the goal to uncover the susceptibility to VTE in patients devoid of major clinical risk factors. Conventional thrombophilia testing typically involves the determination of deficiencies in antithrombin, protein C, or protein S, and genetic testing for the factor V Leiden (FVL) and prothrombin G20210A variants. 3 However, despite their widespread use, these tests are critically flawed with limited sensitivity and specificity for VTE, as well as with methodological issues. Moreover, conventional thrombophilia tests fail to differentiate between patients at high and low risk of VTE recurrence. 4 5 6 Additionally, thrombophilia testing introduces risks, with potential long-term personal consequences, if the information is misinterpreted or inappropriately applied. As a consequence, current guidelines recommend against the routine use of thrombophilia tests. 7 8 9 10 Nonetheless, there is substantial nonadherence to the guidelines in clinical practice. In a recent, large, single-center cross-sectional study, merely one-third of thrombophilia tests were conducted according to the guidelines. 11 Furthermore, the study underscored the limited therapeutic value, with only 8.3% of thrombophilia tests influencing anticoagulant treatment decisions. 11

To underscore the importance of judicious use of thrombophilia tests and to highlight their potential psychological and social consequences, we here present three unrelated cases of genetic discrimination after testing for inherited thrombophilia. The cases were selected from the outpatient clinic of the Thrombosis and Haemostasis Department at the University Hospitals Leuven, Belgium. All three patients presented between 2020 and 2023 after medical disqualification by the police department based on the results of previous thrombophilia tests. To the best of our knowledge, no additional cases of discrimination resulting from thrombophilia testing were documented at our institution. The retrospective study of our cases was approved by the Ethics Committee Research UZ/KU Leuven. Informed consent for publication was obtained from all three patients.

## Case 1

A 22-year-old woman in good overall health externally underwent selective FVL testing after her grandfather was tested heterozygous for FVL following a thrombotic event. Her test revealed that she was also a heterozygous carrier of FVL. She switched from the estrogen–progestogen contraceptive pill to a progestogen-only pill. She has never experienced any thrombotic event.

## Case 2

A 20-year-old man, with an unremarkable medical history besides a tonsillectomy and a traumatic radius fracture, was selectively tested for FVL by his hematologist because of his mother's history of a contraceptive pill-related deep vein thrombosis and positive FVL testing. The results showed heterozygosity for FVL. No specific interventions were undertaken.

## Case 3

A 36-year-old man, with no medical history, underwent thrombophilia testing via his general practitioner after his mother was found to have FVL heterozygosity following an unprovoked deep vein thrombosis. The test showed normal plasma levels for antithrombin, protein C, and protein S, but clear activated protein C (APC) resistance. Further genetic testing verified his heterozygous FVL carriership. Genetic testing for the prothrombin G20210A variant was not performed. He was advised to start thromboprophylaxis in high-risk situations and to maintain increased vigilance toward VTE symptoms.

Despite their overall good medical condition, all three individuals were denied by the medical department of the police academy based solely on their asymptomatic carriership of FVL. The decision was grounded in the perceived higher risk of VTE associated with the combination of FVL and their potential employment as police officers. 12

The decision for medical disqualification is fundamentally unfair. The interpretation of the clinical consequences of a positive thrombophilia test by the police department was incorrect, thereby illustrating the risks and complexities of thrombophilia tests. Moreover, these cases highlight the importance of judicious use of thrombophilia tests, considering the absence of a proper indication for testing according to the most recent 2023 guidelines from the American Society of Hematology (ASH). 10

## Prevalence and Venous Thromboembolism Risk of Factor V Leiden

FVL results from a single missense variant in the *F5* gene (p.Arg534Gln, historically reported as p.Arg506Gln). Hence, a prothrombotic factor V (FV) protein arises, characterized by an increased resistance to inactivation by APC and a loss of FV's anticoagulant cofactor activity in the degradation of FVIIIa by APC. 13 FVL is the most common genetic risk factor associated with VTE, with a prevalence of approximately 5% among Caucasians and approximately 20% in unselected individuals with VTE. 14 15 16 Heterozygosity for FVL is associated with a 4-fold increase in the risk of VTE, and this risk further escalates to a factor eleven in those with homozygosity for FVL. 17 Nevertheless, the absolute risk of VTE remains low, with an annual incidence ranging from 0.45 to 0.67% per year in asymptomatic heterozygous carriers, compared with the overall population incidence of approximately 0.1 to 0.2% per year. 18 19 20 In FVL carriers under 30 years of age, this incidence is even lower at 0.25% per year. 18 19 Ultimately, only 11% of all FVL carriers will develop VTE during their lifetime, with over half of these events being triggered by clinical risk factors such as recent surgery, trauma, or pregnancy. 18 19 21 Thus, FVL should only be considered as a genetic “risk factor” as it is not at all fully penetrant for VTE. The thrombotic risk associated with FVL carriership does not justify routine thromboprophylaxis in asymptomatic carriers, given the increased bleeding risk associated with anticoagulant treatment. When VTE does occur, the treatment is generally uncomplicated, and neither the choice nor the duration of treatment is typically influenced by the presence of FVL. Clinical decision-making regarding VTE therefore relies on clinical factors rather than the presence of hereditary thrombophilia. Consequently, this genetic risk factor on itself is not a reason to declare someone unfit for a profession.

## Guidelines on Thrombophilia Testing

Thrombophilia tests may provide insights into the etiology of (unprovoked) VTE, yet their therapeutic implications are currently limited. Current guidelines therefore recommend against their routine use in clinical practice ( Table 1 ). 7 8 9 10 Instead, thrombophilia tests should only be performed when results will influence management. However, due to a lack of randomized controlled trials, the level of evidence supporting these guidelines is weak. Moreover, guidelines are inconsistent and ambiguous, resulting in poor adherence and misuse in clinical practice. 11 According to the guidelines, thrombophilia tests should not be performed in asymptomatic men with known familial FVL, as in case 1 and 3. 7 8 9 10 Nonetheless, guidelines disagree on testing asymptomatic women with familial FVL, as in case 2 ( Table 1 ).

**Table 1:** Overview of guidelines for thrombophilia testing in clinical practice

	2009 GFHT 7	2016 ACF 8	2022 BSH 9	2023 ASH 10
Primary prevention in patients with family history of VTE				
Known familial thrombophilia				
AT, PC, or PS deficiency	Recommended in first-degree relatives < 60 y ^a^	Only considered in first-degree related women contemplating COC/pregnancy	Recommended in first-degree relatives	Recommended in first- and second-degree relatives with minor VTE risk factors, including postpartum and COC use ^c,d^
Heterozygous FVL or FII G20210A variant	Only recommended in women of childbearing age	Only considered in first-degree related women contemplating COC/pregnancy	Not recommended ^b^	Not recommended ^d^
Unknown familial thrombophilia				
	Not recommended	Not recommended	Not recommended	Not recommended ^d^
Secondary prevention following personal VTE				
Provoked VTE	Recommended in women of childbearing age Recommended in case of recurrent VTE < 60 y	Not recommended	Not recommended	Only recommended when transient nonsurgical risk factors, including postpartum and COC use ^e^
Unprovoked VTE	Recommended in anyone < 60 y	Only recommended if low bleeding risk and plan to stop anticoagulation	Only, recommended when strong personal and/or family history of thrombosis	Not recommended

Abbreviations: ASH, American Society of Hematology; AT, antithrombin; BSH, British Society of Haematology; COC, combined oral contraceptive; FVL, factor V Leiden; PC, protein C; PS, protein S; VTE, venous thromboembolism; ACF, anticoagulation forum; GFHT, French Group of Hemostasis and Thrombosis.

^a^ Also in case of homozygous polymorphisms for FVL and FII G20210A as well as double heterozygotes.

^b^ Prior to COC use in women with a first-degree relative with FVL and history of thrombosis, thrombophilia testing can be discussed case by case.

^c^ Minor provoking risk factors: immobility, minor injury, illness, infection.

^d^ n ambulatory patients with cancer at low or intermediate VTE risk, who have a first-degree relative with VTE, thrombophilia testing is recommended, regardless of known familial thrombophilia.

^e^ Non-surgical risk factors: immobilisation > 3 days, use of COC, pregnancy, post-partum.

The most recent 2023 ASH guidelines recommend thrombophilia testing for patients with symptomatic VTE provoked by transient nonsurgical risk factors, pregnancy, postpartum period, or combined oral contraceptives (COC), to determine the need for lifelong anticoagulation. 10 Indefinite anticoagulation is advised for patients with confirmed thrombophilia. Additionally, thrombophilia testing is advised for asymptomatic individuals with a family history of VTE and known antithrombin, protein C, or protein S deficiency, with the recommendation for thromboprophylaxis in risk situations and avoidance of COC in thrombophilic patients. Nevertheless, thrombophilia testing is not supported for patients with VTE provoked by major risk factors, nor for asymptomatic individuals with a family history of VTE whose familial thrombophilia status is unknown or who have a known heterozygous FVL or prothrombin G20210A variant in the family.

## Risk of Patient Harm by Thrombophilia Tests

Considering not only the clinical implications but also the potential psychological and social ramifications arising from misuse or misinterpretation of thrombophilia tests is paramount in the context of thrombophilia testing ( Table 2 ). According to the 2023 ASH guidelines, all three case patients had no firm indication for thrombophilia testing and would have successfully passed medical examinations if they had not undergone such testing. 10 Nonetheless, discrimination on any grounds, including genetics, is explicitly prohibited, as stated in Article 14 of the European Convention on Human Rights. However, genetic discrimination persists anno 2023 and continues to impact individuals' lives. These instances are not isolated, as evidenced in a survey by Bank et al. among healthy FVL carriers, revealing reports of stigmatization in healthcare and discrimination by insurance companies. 22

**Table 2:** Pros and cons for thrombophilia testing

Pro	Con
May provide insights into VTE susceptibility and improve knowledge and counselling	Limited sensitivity and specificity for VTE • Risk of false reassurance with negative test • Risk of overtreatment with positive test
May prevent first VTE in asymptomatic confirmed thrombophilia cases by e.g., • No estrogen–progestogen contraceptive pill • Extending postpartum thromboprophylaxis	Limited therapeutic implications as clinical decision-making mainly relies on clinical risk factors
	Misinterpretation and misuse • Psychological consequences: worry • Social consequences (discrimination): profession, insurance
	High costs

Abbreviation: VTE, venous thromboembolism.

The emergence of multigenetic thrombophilia tests to screen for (anti-)coagulation defects holds promise for enhancing diagnostic precision and counselling. 23 However, uncertainties persist concerning the clinical usefulness and cost-effectiveness. Furthermore, pervasive genetic testing reveals numerous variants of unknown significance, complicating its use and thereby increasing the risk of misinterpretation or misapplication with potential adverse patient consequences. 24 Future studies are imperative to ascertain the precise place of both conventional and multigenetic thrombophilia testing within clinical practice. Until then, prudence in interpretation and rational use of thrombophilia tests are warranted to prevent potential unnecessary harm to patients.
